# RmmLII, a novel marine-derived *N-*acyl homoserine lactonase from *Tritonibacter mobilis*

**DOI:** 10.3389/fmicb.2025.1538873

**Published:** 2025-03-18

**Authors:** Yu Shen, Dongwei Liu, Xiaoxue Yue, Dongliang Wang, Zhikui Wang, Xu Wang, Gang Liu, Xiaodong Liu, Xiulei Cai

**Affiliations:** ^1^College of Veterinary Medicine, Qingdao Agricultural University, Qingdao, China; ^2^Qingdao West Coast New District People’s Hospital, Qingdao, China

**Keywords:** quorum quenching enzyme, *N*-acylhomoserine lactonase, *Tritonibacter mobilis*, RmmLII, *Pseudomonas aeruginosa* PAO1

## Abstract

**Introduction:**

Quorum sensing (QS) is a bacterial intercellular communication system that can regulate the expression of various virulence genes coordinate the group behaviors of the bacteria by sensing the concentration of signaling molecules in the surrounding environment. An increase in bacterial drug-resistance has been caused by the widespread use of antibiotics, making it urgent to identify safe and effective alternatives to antibiotics. Quorum quenching (QQ) is a strategy to control bacterial infections by disrupting the QS system, which reduces pathogenicity or increases biofilm susceptibility to antibiotics. Several natural agents with QQ activity have been identified, including small molecular inhibitors and QQ enzymes that disrupt bacterial QS by degrading or modifying the QS signal molecules.

**Methods:**

In the present study, We performed heterologous recombinant expression of the potential QQ enzyme-encoding gene *RmmLII* from *Tritonibacter mobilis* YJ3. The degradation activity of RmmLII against AHLs was assessed *in vitro* using the A136 liquid X-Gal assay and a plate detection method. Furthermore, the degradation mechanism of RmmLII was analyzed via high-performance liquid chromatography-mass spectrometry (HPLC-MS). The effects of RmmLII on extracellular proteases production, pyocyanin synthesis, rhamnolipids secretion, biofilm formation, and motility of *Pseudomonas aeruginosa* PAO1 were evaluated in vitro. Additionally, a mouse infection model was established using *P. aeruginosa* PAO1 to investigate the impact of RmmLII on the production of inflammatory cytokines IL-1β, IL-6, and TNF-α, as well as mouse survival rates.

**Results:**

A novel *N-*acylhomoserine (AHL) lactonase RmmLII was identified and characterized from *T. mobilis* YJ3, which was isolated from healthy shrimp in our previous work. Through amino acid sequence alignment, a conserved “HXHXDH” domain was detected in RmmLII, indicating that RmmLII belongs to the phosphotriesterase (PTE) family. Recombinant RmmLII could effectively degrade AHLs *in vitro*, both long-chain and short-chain AHLs, ranging from C_6_ to C_14_. It exhibited the strongest quenching effect on C_6_-HSL, C_8_-HSL, C_10_-HSL, 3-oxo-C_8_-HSL, 3-oxo-C_10_-HSL, 3-oxo-C_12_-HSL, and 3-oxo-C_14_-HSL, while the quenching effect on C14-HSL and 3-oxo-C_6_-HSL was relatively weaker, especially with more notable degradation activity towards long-chain AHLs with a substitution of oxo-group at the C-3 position. HPLC-MS analysis revealed that RmmLII could hydrolyze the ester bond of AHLs. In addition, RmmLII significantly inhibited the production of extracellular proteases, pyocyanin, rhamnolipids, biofilm formation, as well as motility of *P. aeruginosa* PAO1 *in vitro*. It also reduced the production of inflammatory factors IL-1β, IL-6, and TNF-α, thereby improving the survival rates of mice infected with PAO1 *in vivo*.

**Discussion:**

This study demonstrates the potential application of RmmLII in controlling PAO1 infections, offering new insights for the development of novel antibiotic alternatives. RmmLII has the potential as a therapeutic agent for application in the mitigating PAO1 infections.

## 1 Introduction

Quorum sensing (QS) serves as a vital communication mechanism utilized by bacteria, fungi, and phages (Mukherjee and Bassler, 2019). Bacteria evaluate population density by detecting the concentrations of signaling molecules, which regulate gene expression and coordinate group behavior within bacterial populations. The regulations of bacterial physiological functions by the QS system primarily includes the following aspects: biofilm formation (Engebrecht et al., 1983), virulence factor expression (Bronesky et al., 2016), and motility (Engebrecht and Silverman, 1984). The principal signaling molecules recognized within the QS system comprise *N-*acylhomoserine lactones (AHLs) (Galloway et al., 2011), autoinducer-2 (AI-2) (Lowery et al., 2008), and autoinducing peptides (AIPs) (West et al., 2021). In gram-negative bacteria, the AHLs are important for QS induction (Remy et al., 2018), which triggers the expression of genes that modulate bacterial behavior and facilitate interactions within communities. In *Pseudomonas aeruginosa*, there are at least three QS systems, namely those mediated by *Las*, *Rhl*, and *Pqs, respectively.* These QS systems play a crucial role in regulating the virulence phenotypes and the production of virulence factors (Rather et al., 2022).

Quorum quenching (QQ) is a strategy to counteract the QS-regulated phenotypes, such as virulence expression and biofilm formation, by interfering with QS. On the basis of the functional targets, QQ agents usually work by inhibiting the synthesis of AIs, degrading and inactivating AI signaling molecules, interrupting AI signal receptors, inhibiting AIs efflux, and blocking the downstream signaling cascades (Rather et al., 2022). The QQ agents can be divided into two groups, QS inhibitors and QQ enzymes, based on the molecular weight. QQ enzymes can interfere with QS by degrading extracellular signals, such as AHLs, thereby regulating the expression of virulence genes (Bzdrenga et al., 2017). This disruption inhibits bacterial pathogenicity and contributes to disease prevention and control. Consequently, there is a growing interest in the use of QQ enzyme technology to interfere with bacterial QS systems as a strategy for mitigating infections. AHL-degrading enzymes can be categorized into three types, AHL lactonases, AHL acylases, and AHL oxidoreductases according to their enzymatic mechanisms (Tang and Zhang, 2014). AHL lactonase is the main type of AHL-degrading enzyme, which can produce the corresponding *N*-acyl homoserine by hydrolyzing the lacone ring of AHL molecules. To date, approximately 30 AHL lactonases have been reported, which are primarily associated with the metallo-β-lactamase (MBL) superfamily, the phosphotriesterase (PTE) family, and the α/β hydrolase family (Hao et al., 2024; Ryu et al., 2020). The first identified AHL lactonase, AiiA from *Bacillus* sp. strain 240B1 (Dong et al., 2000), belongs to the MBL superfamily and contains a conserved “HXHXDH” domain. Since then, an increasing number of lactonases have been reported. For instance, SsoPox (Ng et al., 2011) from *Sulfolobus solfataricus*, a lactonase resembling phosphotriesterases, significantly reduced the production of virulence factors such as elastase, protease, and pyocyanin in *P. aeruginosa* PAO1. MomL (Tang et al., 2015) from *Gordonia* sp. Th120 has been reported to exhibit strong activity in reducing the virulence of *P. aeruginosa* PAO1 in the *Caenorhabditis elegans* infection model, which further supports that QQ is a promising anti-virulence strategy. Thus, QQ enzyme technology is a potential strategy to interfere with bacterial infections, especially those caused by multidrug resistant bacteria.

*Tritonibacter* species, the members of the Roseobacter group, are widely distributed in marine microbial communities (Wagner-Dobler and Biebl, 2006; Wirth and Whitman, 2018). These bacteria are abundant and capable of inhibiting the growth of various pathogens, making them valuable marine-derived beneficial bacteria. The known members of the Roseobacter group produce various antibacterial substances, including tropodithietic acid (TDA) (Cude et al., 2012), indigoidine (D'Alvise et al., 2010), Roseochelin B (Wang and Seyedsayamdost, 2017), and methyltroposulfenin, among others (Sonnenschein et al., 2021). In our previous study, we demonstrated that *Tritonibacter mobilis* YJ3, isolated from a healthy shrimp, has strong AHL-degradative activity and could synthesize AHL lactonase RmmL (Cai et al., 2018). In this study, another novel AHL lactonase, RmmLII, was identified in *T. mobilis* YJ3, and its enzymatic properties were characterized. In addition, interference with the QS system of *P. aeruginosa* PAO1 was examined both *in vitro* and *in vivo*. This study is expected to establish a foundational framework for investigating the application of QQ enzymes in controlling of bacterial diseases, while also demonstrating the potential of QQ enzymes as innovative antibacterial agents for attenuating the pathogenicity of such infections in both veterinary and human medicine.

## 2 Materials and methods

### 2.1 Bacterial strains, media, growth conditions and chemicals

*T. mobilis* YJ3 was cultured in 2216E marine broth (MB, Becton Dickinson, Franklin Lakes, NJ, United States) at 28°C. The AHL biosensor *Agrobacterium tumefaciens* A136 (pCF218) (pCF372) was maintained on Luria Bertani (LB) agar and grown in AT minimal medium containing 0.5% (wt/vol) glucose for detecting AHLs (C_6_ to C_14_) by the liquid X-Gal (5-bromo-4-chloro-3-indolyl-β-D-galactopyranoside) assay (Tang et al., 2013). The AHL biosensors *Chromobacterium violaceum* CV026 and VIR24 (Latifi et al., 1995) were used for detecting short-chain (C_4_ to C_8_) and long-chain (C_8_ to C_14_) AHLs, respectively, by culturing them on LB agar plates and incubating at 28°C. *Escherichia coli* BL21 (DE3) was cultured in LB medium supplemented with kanamycin (50 μg/mL) and incubated at 37°C, it served as the host for expressing proteins whose encoding genes were constructed into the pET28a(+); *P. aeruginosa* PAO1 was cultured in nutrient broth (NB) medium at 37°C.

C_6_-HSL, 3-oxo-C_6_-HSL, C_8_-HSL, and C_14_-HSL were purchased from Cayman Biologicals (Ann Arbor, MI, USA); 3-oxo-C_8_-HSL, C_10_-HSL, 3-oxo-C_10_-HSL, C_12_-HSL, 3-oxo-C_12_-HSL, 3-oxo-C_14_-HSL, Azocasein, Orcinol (3,5-dihydroxytoluene) were purchased from Sigma Chemical (St. Louis, MO, USA); all AHLs were diluted by dimethyl sulfoxide (DMSO) into 10–500 mM and stored at −20°C. Enterokinase was purchased from Sangon Biotech (Shanghai) Co., Ltd. Chloroform was purchased from Sinopharm Chemical Reagent Co., Ltd.

### 2.2 Cloning, expression, and purification of RmmLII

Experimentally validated protein sequences of active QQ enzymes were retrieved from the UniProt online database^1^ for the purpose of constructing a local database. The putative lactonase gene *rmmLII* from *T. mobilis* YJ3 was searched from the whole-genome sequence of YJ3 (Genbank accession No. CP181340-CP181345) through local BLASTP analysis against some known QQ enzyme sequences. The amino acid sequences of verified AHL lactonases were systematically compared to RmmLII by using the sequence analysis software MEGA11, while the Neighbor-Joining (NJ) method was employed to construct a phylogenetic tree. The results derived from the multiple sequence alignment were presented through ESPript, along with a detailed analysis of the active site residues.

The genomic DNA of *T. mobilis* YJ3 was extracted by the phenol-chloroform method, and *rmmLII* was amplified by the PCR method using a pair of primers: *rmmLII*-F: 5′-CGC GGA TCC GAC GAC GAC AAG ATG GTG TCG GTG GCG-3′ and *rmmLII*-R: 5′-CCC AAG CTT CTA GCG GCG CTG GTA AAC-3′, and then cloned into pET28a(+) to construct a recombinant plasmid pET28a(+)-*rmmLII*. The restriction enzyme sites *BamH* I and *Hind* III were incorporated into the forward and reverse primers, respectively, and an EK enzyme site was inserted to remove the His tag from the purified protein. PCR was conducted using the following conditions: denaturation at 98°C for 30 s, followed by 35 cycles of denaturation at 98°C for 15 s, annealing at 55°C for 30 s, and extension at 72°C for 90 s.

The recombinant plasmid pET28a(+)-*rmmLII* was then transformed into *E. coli* BL21 (DE3) to construct the recombinant strain *E. coli* BL21 (DE3)/pET28a(+)-*rmmLII* for protein expression. The bacterial strain was cultured in LB liquid medium added to 30 μg/mL Kan at 37°C with shaking at 170 rpm until the OD_600_ of the culture was 0.4–0.6. Subsequently, 0.1 mM IPTG (isopropyl-β-D-thiogalactopyranoside) was added to the culture induce protein expression, and the culture was incubated at 16°C with moderate shaking (150 rpm) for 15 h. The induced culture was subjected to high-speed centrifugation at 4°C to pellet the cells, which were then washed once with PBS buffer (50 mM, pH 7.0). The cells were resuspended with 10 times the original volume of binding buffer (Tris–HCl, 10 mM; imidazole, 10 mM; NaCl, 0.5 M). The cell suspension was sonicated on an ice bath, and the supernatant was collected via centrifugation at 13,000 rpm for 10 min at 4°C and filtered through a 0.22-μm PVDF membrane. The sample was loaded onto the NTA-Ni (Qiagen) column for purification, following the manufacturer’s instructions, to obtain the recombinant RmmLII-His fusion protein. In order to remove the His tag from purified protein, RmmLII-His was incubated overnight at 4°C with enterokinase, and then purified again by NTA-Ni columns. The purified RmmLII was analyzed by SDS-PAGE and stored in Tris–HCl buffer containing 10% glycerol at −20°C.

### 2.3 Activity assay of RmmLII against different AHLs

The degradation activity of RmmLII on AHLs was tested under the A136 liquid X-Gal method described previously (Tang and Zhang, 2018). Briefly, the A136 reporter strain was cultured in LB broth (containing spectinomycin 50 μg/mL, tetracycline 4.5 μg/mL) at 28°C until the OD_600_ reached approximately 2.0. RmmLII, 1,4-piperazinediethanesulfonic acid (PIPES) buffer (1 mM, pH 6.7), and various AHL signal molecules (1 mM) were mixed in a volume ratio of 10:1:0.1 and incubated at 28°C for 24 h. Tris–HCl was used as a substitute for RmmLII in the negative control. A 1% solution of A136 was added to the AT medium (containing spectinomycin 50 μg/mL, tetracycline 4.5 μg/mL) with a final concentration of 250 μg/mL of X-Gal. The mixture was thoroughly mixed to obtain the detection solution. Then 10 μL of the reaction supernatant was combined with 190 μL of the detection solution in a 96-well plate and incubated at 28°C for 24 h. The OD_492_ and OD_630_ were measured, and the β-galactosidase activity values were determined, which were inversely correlated with the RmmLII enzymatic activity. Based on Equation (1), the calculation was performed accordingly.


(1) $$\begin{array}{l} \beta -\mathrm{galactosidase activity}\\ =\left[\left(0.653\times {OD}_{492}–{OD}_{630}\right)/\left(0.267\times {OD}_{630}–{OD}_{492}\right)\right] \end{array}$$

To further confirm the degradation activity of RmmLII, a plate detection method was employed. *C. violaceum* CV026 and VIR24 were inoculated into LB broth and incubated overnight at 28°C. Then, the cultures were introduced into the semi-solid LB medium consisting of 0.8% (wt/vol) agar at approximately 45°C with an inoculum amount of 3%. After the medium solidified, the wells were created, and the reaction solution was added to each well. The plates were incubated at 28°C for 24 h to observe the formation of blue or purple halos and size.

### 2.4 High-performance liquid chromatography-mass spectrometry analysis of products formed by RmmLII catalysis

To determine the chemical structure of the degradation products resulting from AHL signaling molecule catalyzed by RmmLII, 10 μL of RmmLII (1 mg/mL) was mixed with 100 μL of PIPES buffer (1 M, pH 6.7), 200 μL of C_10_-HSL (10 mM), and deionized water was added up to a final volume of 1 mL. The mixture was thoroughly mixed and incubated at 28°C for 24 h, and was extracted thrice by ethyl acetate. The ethyl acetate solution was then evaporated to dryness by a nitrogen blower and analyzed by high-performance liquid chromatography (HPLC) using a SunFire C18 reversed-phase column (3.5 μm, 4.6 by 50 mm) using a mobile phase of acetonitrile-water (0.01% trifluoroacetic acid; a linear gradient [v/v] of acetonitrile from 5 to 95% over 15 min, held for 1 min, and then reduced to 5%; flow rate of 0.5 mL/min). The resulting fractions were analyzed by electrospray ionization mass spectrometry (ESI-MS).

### 2.5 Site-directed mutagenesis of RmmLII

To investigate the effect of specific amino acids on the activity of RmmLII, site-directed mutagenesis was performed using the Fast Mutagenesis System kit (TransGen, China) according to the manufacturer’s instructions. Briefly, the pET28a(+)-*rmmLII* plasmid was used as the template for mutagenesis. The primers used to obtain mutants are listed in Table 1. The mutated plasmids were sent to Sangon Biotech for sequencing to confirm the accuracy of the desired mutation sites and then transformed into *E. coli* BL21 (DE3) competent cells. The expression and purification of each mutated protein were conducted as described above, and the activities of mutated proteins were tested by the A136 liquid X-Gal method and compared to that of purified RmmLII.

**Table 1:** Primers used in mutagenesis.

Site-directed	Primer-F (5′-3′)	Primer-R (5′-3′)
P82H	CGCTGGGTGGCAAACACGTGCGCCGTGTC	GTGTTTGCCACCCAGCGGACCCGCCATCAG
A116E	CGTGACCACACGCACCGAGTGGCTTTTTGC	CTCGGTGCGTGTGGTCACGAGCTCTGCGC
E198G	ACGGTCATGCGCCGGGACACGCAACCCTC	TCCCGGCGCATGACCGTTGCCCATGTGGA
E241D	GCTGGCGGAATGGCTGGACGCCTGCGAAC	GTCCAGCCATTCCGCCAGCGGGTCGGCCTC

Mutagenic codons are underlined.

### 2.6 Physical and chemical parameters that affect RmmLII activity

The optimal temperature, thermostability, pH stability and the effects of various metal ions on the activity of RmmLII (1 mg/mL) were measured by the A136 liquid X-Gal method using C_6_-HSL as the substrate. Each experimental group was performed with a minimum of three independent replicates to ensure reproducibility and statistical reliability.

The reaction solution was prepared according to the method described in Section 2.3. For the optimal temperature, the prepared reaction solution was incubated at temperatures of 10°C, 20°C, 40°C, 50°C, 60°C, 70°C, 80°C, 90°C, and 100°C for a duration of 24 h. Following incubation, OD_492_ and OD_630_ were measured, and β-galactosidase activity was calculated using Equation (1), with the activity of RmmLII incubated at 28°C used as the control. The relative activity under other conditions was calculated with this as 100%. To determine the thermostability of RmmLII, purified RmmLII was preincubated at temperatures of 20°C, 40°C, 60°C, 80°C, and 100°C for 30 min, after which the residual activity was evaluated. The β-galactosidase activity values of untreated RmmLII reactions incubated at 28°C were employed as the control values. Similarly, the pH stability was tested after the purified RmmLII was preincubated at pH levels ranging from 2 to 11 at 4°C for 3 h. To evaluate the impact of different metal ions, 1 mM of EDTA was combined with 0.1 mM of metal ions (Zn^2+^, Mg^2+^, Fe^2+^, Ca^2+^, Cu^2+^, Mn^2+^) and added to purified RmmLII, which was then incubated at 4°C for 1 h. Subsequently, metal ions were added to the EDTA-treated RmmLII at a concentration of 1.1 mM, and the mixture was incubated at 4°C for an additional 1 h. The residual activity of RmmLII was subsequently evaluated, with untreated RmmLII serving as the control.

### 2.7 Effect of RmmLII on virulence factors production in *Pseudomonas aeruginosa* PAO1 *in vitro*

To evaluate the impact of RmmLII on the production of virulence factors in *P. aeruginosa* PAO1, PAO1 was cultured overnight together with various concentrations of RmmLII [low dose group (1 × 10^−3^ mg/mL), medium does group (3.75 × 10^−3^ mg/mL), high dose group (7.5 × 10^−3^ mg/mL)] in LB or PB medium at 37°C, PAO1 culture without RmmLII was used as the negative control. The secretion levels of virulence factors, including extracellular protease, pyocyanin, and rhamnolipid, were assessed. Each experiment was performed in triplicate.

#### 2.7.1 Pyocyanin

For pyocyanin measurement, a method adapted from a previous study (Abdelaziz et al., 2023; Najafi et al., 2021) was used. *P. aeruginosa* PAO1 was cultured in PB medium (20 g/L peptone, 1.4 g/L MgCl_2_, 10 g/L K_2_SO_4_, 10 mL glycerol) at 37°C until pyocyanin production was achieved. The cultures were then transferred into PB medium containing different concentrations of RmmLII at an inoculation rate of 1% (v/v). The culture without RmmLII served as the negative control. All cultures were incubated at 37°C with constant agitation at 200 rpm for 24 h. Subsequently, 5 mL of the culture solution was mixed with 3 mL of chloroform and extracted three times. After centrifugation at 12,000 rpm for 2 min, 200 μL of the lower chloroform phase was combined with 600 μL of 200 mM HCl and vortexed thoroughly, followed by repeat centrifugation under the same conditions. The upper phase was collected, subjected to a two-fold dilution, and mixed well before measuring OD_520_.

#### 2.7.2 Extracellular protease

The extracellular protease levels were determined by using a method adapted from Hentzer et al. (2002). The activated PAO1 culture was inoculated at a 1% concentration into NB medium containing different concentrations of RmmLII, with no RmmLII added as the control. The samples were incubated at 37°C with shaking at 200 rpm for 24 h, followed by measurement of OD_600_. The culture was centrifuged at 12,000 rpm and 4°C for 10 min to pellet the bacteria, 150 μL of the supernatant was mixed with 250 μL of azocasein (2%, 5 mg/mL in 50 mM Tris–HCl, pH 7.9;), and incubated at 4°C for 4 h. The reaction was terminated by adding 1.2 mL of 10% trichloroacetic acid, followed by incubation at room temperature for 15 min. After centrifugation at 10,000 rpm for 10 min, the supernatant was mixed with an equal volume of 525 mM NaOH and measured the OD_440_. The extracellular protease activity of each sample was calculated using OD_440_/OD_600_.

#### 2.7.3 Rhamnolipid

The rhamnolipid levels were measured following a protocol by July Fong et al. with modifications (Fong et al., 2018). Overnight cultures of PAO1 were diluted to an OD_600_ of 0.01 by NB medium. Different concentrations of RmmLII were added into the diluted cultures, while an untreated control group was maintained under identical conditions. The cultures were incubated overnight at 37°C with shaking at 200 rpm. Following incubation, aliquots of supernatant were collected and centrifuged at 8,000 rpm for 10 min to remove bacterial pellets. The supernatant was acidified to pH 2.0 using HCl, followed by extraction with diethyl ether for three times, and the organic phases were combined and evaporated. The residues were re-suspended in deionized water and mixed with 0.19% (w/v) orcinol solution (dissolved in 50% H_2_SO_4_). The reaction mixture was incubatd in a water bath at 80°C for 30 min, followed by cooling to room temperature for 15 min prior to measure the OD_421_.

### 2.8 Effect of RmmLII on the biofilm production and motility of *Pseudomonas aeruginosa* PAO1 *in vitro*

To assess the inhibitory effect of RmmLII on biofilm formation by *P. aeruginosa* PAO1, a method adapted from Alam et al. (2020) was used. Briefly, *P. aeruginosa* PAO1 was inoculated into LB broth at a 2% concentration and grown to an OD_600_ of 0.3. The culture was then transferred to 24-well plates containing sterile slides, and varying concentrations of RmmLII were added to the same. The plates were then incubated statically at 37°C for 24 h. The samples were then washed twice with sterile PBS buffer, fixed in methanol for 15 min, air-dried, and stained with 0.1% crystal violet for 30 min. Excess dye was removed, and the biofilm was treated with 33% (w/v) acetic acid solution, and mixed well before measuring OD_550_. Biofilm morphology was examined by a Sharp Corporation JSM-7500F scanning electron microscope (SEM) after fixing, dehydrating, drying, and gold coating the samples.

The motility of swimming and swarming of PAO1 was analyzed according to the previous report (Wang et al., 2021). The swimming assay was tested in a medium containing 10 g/L tryptone (Difco), 5 g/L NaCl, and 3 g/L agarose (GIBCO/BRL), which was covered with cling film to prevent desiccation. The swarming assay medium comprised 0.5% (wt/vol) Difco Bacto agar, 8 g/L NB, and 5 g/L glucose (Ling et al., 2010). The sterilized media for both assays was mixed with RmmLII at approximately 45°C. Once solidified, 2 μL of the 1% PAO1 culture was inoculated onto the agar plates. After the medium had fully absorbed, the plates were incubated at 30°C for 12–14 h to observe bacterial motility by mearsuring the diameter of the microcolony. Each experiment was performed in triplicate and then the statistical analysis of the data was performed.

### 2.9 Effect of RmmLII against the pathogenicity of PAO1 *in vivo*

A total of 40 female BALB/c mice, approximately 2 months old and weighing approximately 30 g, were obtained from Qingdao Daren Fucheng Animal Husbandry Co. Ltd. The mice were housed with free access to food and water in a room maintained at 20–22°C. After a 1-week acclimatization period, the experiments were initiated. All animal procedures adhered to the guidelines established by the Animal Ethics Committee of Qingdao Agricultural University (approval number: QAU2023122502).

#### 2.9.1 Experimental plan and sample collection

A total of 40 mice were randomly assigned to four groups: blank control, RmmLII, PAO1 model, and RmmLII+PAO1, with 10 mice in each group. The activated PAO1 strain was cultured at 37°C with shaking at 170 rpm, and the OD_600_ was monitored to ensure the bacterial culture reached the logarithmic growth phase. The PAO1 culture was centrifuged at 4,000 rpm for 5 min at 4°C to harvest the viable bacteria. The bacterial pellets were washed three times with sterile PBS and then diluted to a final concentration of 2 × 10^8^ colony-forming units (CFU/mL). A gradient pre-test (0.01 mg/mL, 0.1 mg/mL, and 1 mg/mL) was conducted and the results revealed that the concentration of 1 mg/mL most effectively demonstrated degradation activity against AHL signal molecules. Therefore, the concentration of 1 mg/mL was selected for our animal experiments.

The mice were anesthetized with ether, and 100 μL of saline was administered intranasally to the blank control, RmmLII, and PAO1 model groups. After a 1 min retention period, the mice in the RmmLII+PAO1 group were pre-treated with 100 μL of RmmLII (1 mg/mL) using the same method. After a 12 h interval, the RmmLII group received an additional 100 μL of RmmLII (1 mg/mL). The PAO1 and RmmLII+PAO1 groups were treated with 100 μL of the PAO1 solution 2 × 10^8^ CFU/mL, while the blank control group received an equal volume of saline. The mice were monitored for disease onset throughout the study. The blood samples were collected at 6 h, 12 h, and 24 h post-infection from three randomly selected mice per group at each time point. Before euthanasia via cervical dislocation, the mice were anesthetized with ether, and blood was drawn from the orbital venous plexus. The lung tissues were harvested under sterile conditions.

#### 2.9.2 Enzyme-linked immunosorbent assay for the serum levels of various cytokines

Blood sample is more easier to obtained and standardized in animal experiments. During bacterial infection, cytokines level of the blood can reflect the overall systemic immune response (Valaperti et al., 2019). Mice blood samples were obtained and incubated at 37°C for 1 h to allow serum separation. After incubation, the samples were centrifuged at 3,000 rpm for 10 min to obtain the upper yellowish serum layer, which was stored at −80°C until further analysis. The serum samples were thawed at 4°C for 20 min and subsequently equilibrated at room temperature for 40 min before testing. Tumor necrosis factor-α (TNF-α), interleukin-6 (IL-6), and interleukin-1β (IL-1β) ELISA kits were purchased from Shanghai Enzyme-linked Biotechnology Co., Ltd., Shanghai, China, and the experiment was conducted in accordance with the manufacturer’s instructions.

#### 2.9.3 Histological analysis

The lung tissues from the lower lobe of the left lung of mice were excised 24 h post-infection, rinsed with saline, and fixed in 4% paraformaldehyde for 48 h. Following fixation, the tissues undergo dehydration and paraffin embedding, after which they were sectioned into 3–5-μm-thick slices and subsequently stained with hematoxylin and eosin (HE) for microscopic observation.

#### 2.9.4 Lung bacterial load count

The lung tissue samples were collected aseptically, rinsed with 0.9% NaCl solution, and weighed. The tissues were diluted to a concentration of 1 mg/mL with 0.9% NaCl solution and then homogenized with grinding steel beads in a tissue grinder. The homogenate was diluted, spread onto Nalidixic Acid Cetrimide agar (NAC) plates, and the plates were cultured in 37°C for 12 h. The colonies were then counted to determine CFU/mg lung tissue, with triplicate samples analyzed for each group (Zhuang et al., 2022).

### 2.10 Statistical analysis

Data are presented as mean ± standard deviation (SD) from at least three independent experiments. Statistical analysis was performed using GraphPad Prism 8 software. Comparisons between two groups were made using the Student’s *t*-test, while multiple comparisons were conducted using one-way ANOVA. Statistical significance was indicated as follows: ns (not significant), *p < 0.05, **p < 0.01, ***p < 0.001, and ****p < 0.0001.

## 3 Results

### 3.1 Amino acid sequence analysis of RmmLII

A local BLASTP search against a known QQ enzyme database identified the QQ enzyme gene Peg3631 in YJ3. This gene encodes a protein of 348 amino acids with high similarity to known AHL lactonases. Phylogenetic analysis of validated AHL lactonases and Peg3631 protein sequences revealed that Peg3631 clusters with the phosphotriesterase family of GKL, demonstrating 58% homology (Supplementary Figure S1). Sequence analysis further indicated that Peg3631 contains a highly conserved “HXHXDH~100AA ~ H” domain characteristic of lactonases (Supplementary Figure S1), suggesting that Peg3631 may represent a novel AHL lactonase and it was named RmmLII.

### 3.2 Expression, purification, and activity test of RmmLII in YJ3

**Figure 1:**
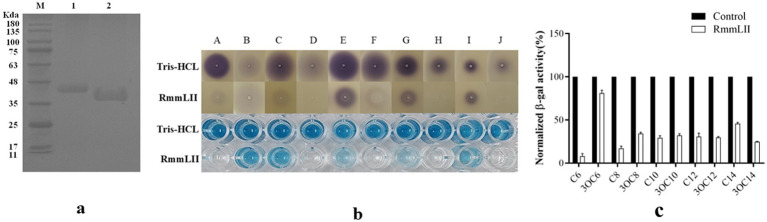
The SDS-PAGE analysis and activity test of purified protein. **(a)** SDS-PAGE analysis of the affinity column-purified RmmLII-His (44.3 kDa, lane 1) and RmmLII protein (39.23 kDa, lane 2) after Trx-tag removal by enterokinase. **(b)** AHLs degradative activity test of RmmLII. The AHL degradative activity of RmmLII was analyzed by the A136 liquid X-Gal assay and Agar plate assay. Tris–HCL used as negative controls, The wells: A, C_6_-HSL; B, 3-oxo-C_6_-HSL; C, C_8_-HSL; D, 3-oxo-C_8_-HSL; E, C_10_-HSL; F, 3-oxo-C_10_-HSL; G, C_12_-HSL; H, 3-oxo-C_12_-HSL; I, C_14_-HSL; J, 3-oxo-C_14_-HSL. **(c)** β-galactosidase activity of RmmLII.

The recombinant plasmid pET28a(+)-*rmmLII* was successfully constructed and transformed into *E. coli* BL21 (DE3) cells for expression. Although more than half of the recombinant proteins were expressed in a soluble form, inclusion bodies were also observed (Supplementary Figure S2). Both purified RmmLII-His and RmmLII proteins displayed a single band corresponding to their predicted molecular weights (Figure 1a). The A136 assay and plate assay results demonstrated that the purified RmmLII exhibits degradation activity toward both short- and long-chain AHLs (ranging from C_6_ to C_14_), with particularly high efficiency against long-chain AHLs containing an oxo-group substitution at the C-3 position (Figures 1b,c). RmmLII showed significantly higher degradation efficiency toward C_6_-HSL, C_10_-HSL, C_12_-HSL, 3-oxo-C_8_-HSL, 3-oxo-C_10_-HSL, 3-oxo-C_12_-HSL and 3-oxo-C_14_-HSL compared to C_8_-HSL, C_14_-HSL and 3-oxo-C_6_-HSL.

### 3.3 HPLC-MS confirm RmmLII as an AHL lactonase

**Figure 2:**
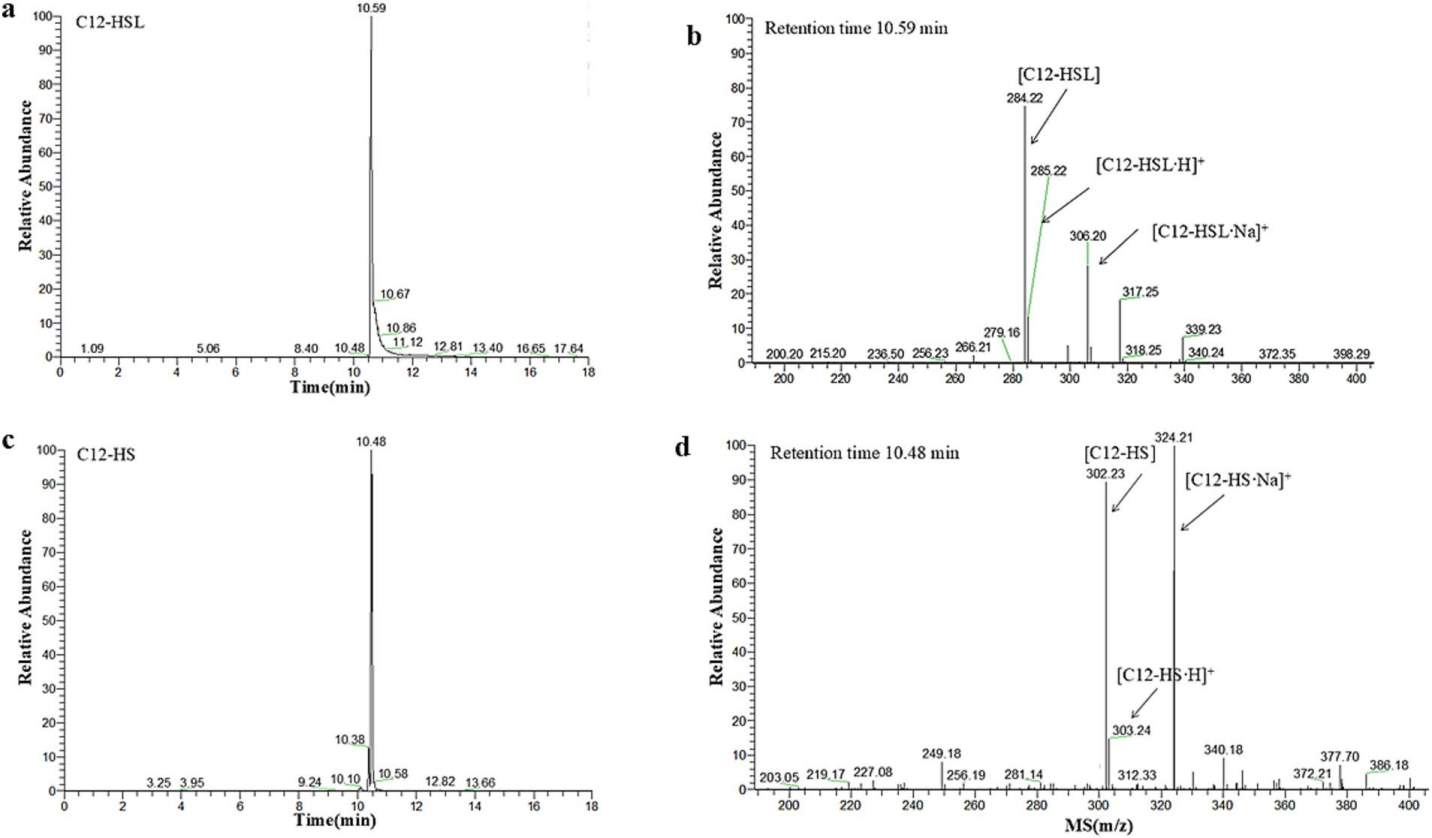
HPLC-MS analysis of the RmmLII-hydrolyzed C_12_-HSL product. **(a)** HPLC profile of the RmmLII-hydrolyzed C_12_-HSL product. **(b)** ESI-MS analysis of HPLC fractions containing the 10.52 min product. **(c)** HPLC chromatogram of the hydrolysis product C_12_-HS. **(d)** ESI-MS analysis of HPLC fractions containing the 10.48 min product.

To gain a deeper understanding of the degradation mechanism of RmmLII, the final degradation products from the reaction between RmmLII and the most effective substrate C_12_-HSL were analyzed using HPLC-MS (Figure 2). The degradation products of C_12_-HSL by RmmLII were detected in liquid chromatography, showing retention times of 10.48 min and 10.52 min. Mass spectral analysis revealed a prominent excimer (M-H) ion at a mass-to-charge ratio (m/z) of 302.23, indicating that the interaction between RmmLII and C_12_-HSL (M-H ion m/z of 284.22) resulted in a mass increase of 18. This increase corresponds precisely to the addition of one water molecule. AHL lactonases are known for their ability to selectively hydrolyze the lactone bond of the signaling molecule (Shen et al., 2021), which aligns with the observed degradation mechanism. Therefore, the peak at a retention time of 10.48 min in the LC represents the degradation product C_12_-HS, confirming RmmLII as an AHL lactonase.

### 3.4 Mutations in the active site amino acids alters the activity of RmmLII

**Figure 3:**
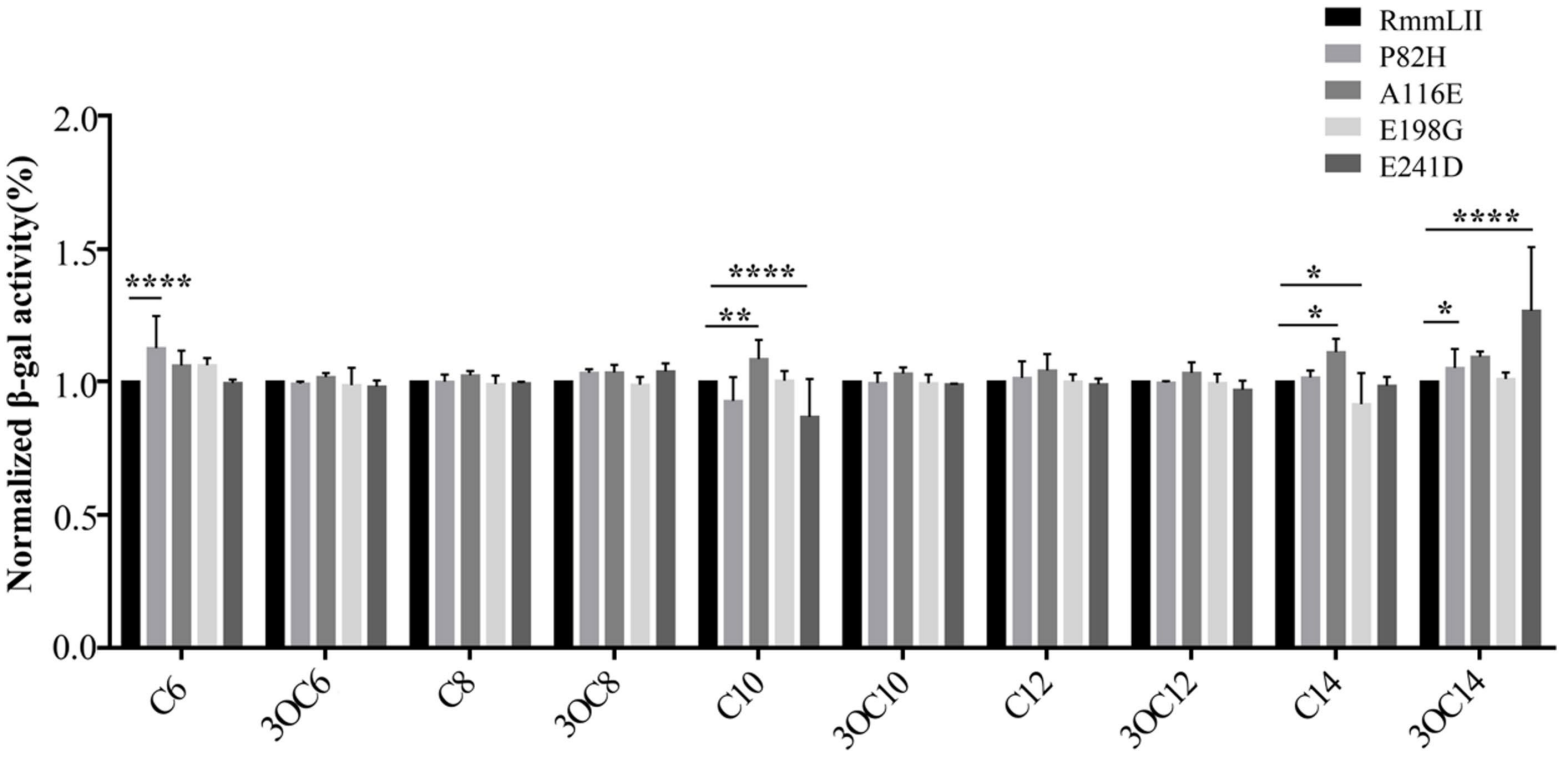
QQ activity of recombinant mutant proteins. Values are reported as the mean ± SD, *n* = 3. *p < 0.05; **p < 0.01; ***p < 0.001; ****p < 0.0001.

To investigate the impact of several conserved and functional amino acids of RmmLII, four selected amino acids of RmmLII were subjected to site-directed mutagenesis and their enzymatic activities were evaluated following protein purification (Figure 3). The residue Pro82 of RmmLII was replaced by Histidine, and the mutated protein (P82H) showed decreased activity toward C_6_-HSL. When the Ala116 was replaced by Glutamic acid, the mutated protein (A116E) exhibited reduced activity against C_10_-HSL, C_14_-HSL, and 3-oxo-C_14_-HSL. Conversely, the mutated protein E198G (Glu198 was replaced by Glycine) demonstrated enhanced degradation of C_14_-HSL. The E241D mutant (Glu241 was replaced by Aspartic acid) displayed increased activity toward C_10_-HSL, reduced activity toward C_14_-HSL, and no significant changes in activity toward other signaling molecules compared to controls. These findings underscore the crucial roles of residues Pro82, Ala116, and Glu241 in RmmLII activity, affecting its degradation capability. The mutation protein (E198G), which enhances C_14_-HSL degradation, suggests that substituting glutamate at position 198 with Glycine may improve degradation capability. These results further emphasize the critical role of these sites in the degradation of AHL signaling molecules, although the exact mechanism remains unclear and warrants further investigation into the relationship between protein structure and function.

### 3.5 Enzymatic characterization of RmmLII

**Figure 4:**
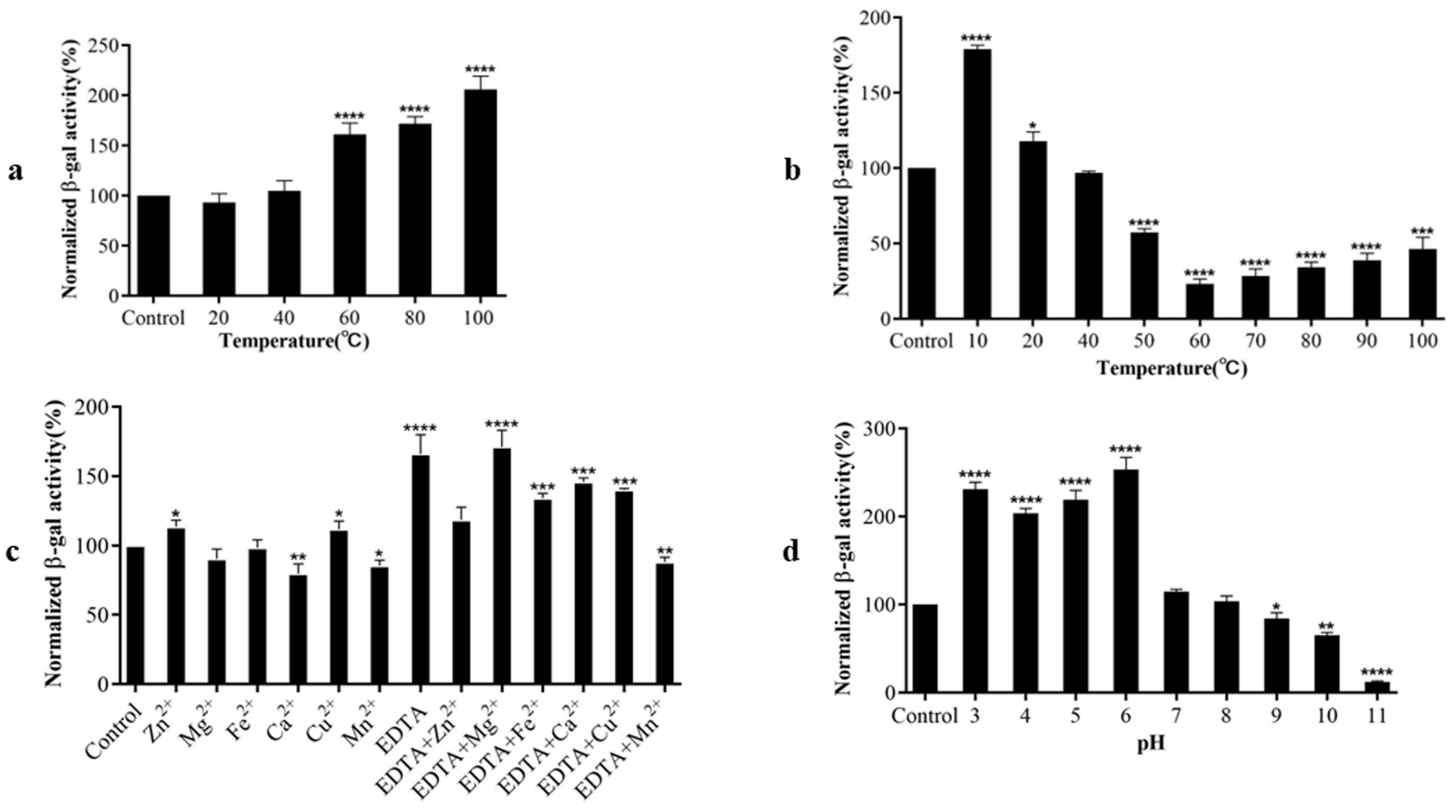
Biochemical characterization of RmmLII activity. **(a)** Temperature tolerance; **(b)** Optimum reaction temperature; **(c)** Effect of different metal ions with EDTA on RmmLII; **(d)** pH. *p < 0.05; **p < 0.01; ***p < 0.001; ****p < 0.0001.

We assessed the influence of various factors, including temperature, pH, EDTA, and metal ions, on RmmLII activity by A136 liquid X-Gal method, in which the β-galactosidase activity is inversely correlated with the activity of RmmLII. After exposure of RmmLII to various temperatures for 30 min, the RmmLII retained stable enzymatic activity between 20°C and 40°C, showing no significant difference compared to the control group (*p* > 0.05). However, the enzyme activity decreased significantly at 60°C–100°C (*p* < 0.0001), culminating in nearly complete inactivation at 100°C (Figure 4a). Following the reaction of RmmLII at various temperatures, high activity was observed within the range of 20–100°C, with enzyme activity peaking at 60°C, identified as the optimal reaction temperature (Figure 4b). After RmmLII was treated with EDTA and various metal ions separately, Zn^2+^ and Cu^2+^ significantly inhibited enzyme activity (*p* < 0.05), while EDTA alone notably reduced RmmLII activity (*p* < 0.0001), and Ca^2+^ and Mn^2+^ enhanced its activity. When various metal ions were added to EDTA-treated RmmLII, EDTA+Mn^2+^ significantly enhanced RmmLII activity (*p* < 0.01), indicating that EDTA forms a stable complex with Mn^2+^, thereby mitigating its partial inhibitory effect on the enzyme (Figure 4c). The enzyme activity of RmmLII significantly decreased within the pH range of 3–6 (*p* < 0.0001), exhibited minimal variation between pH 7–9, and gradually increased at pH > 10, while peak activity was observed at pH 11 (Figure 4d).

### 3.6 Effect of RmmLII on virulence factors production in *Pseudomonas aeruginosa* PAO1 *in vitro*

**Figure 5:**
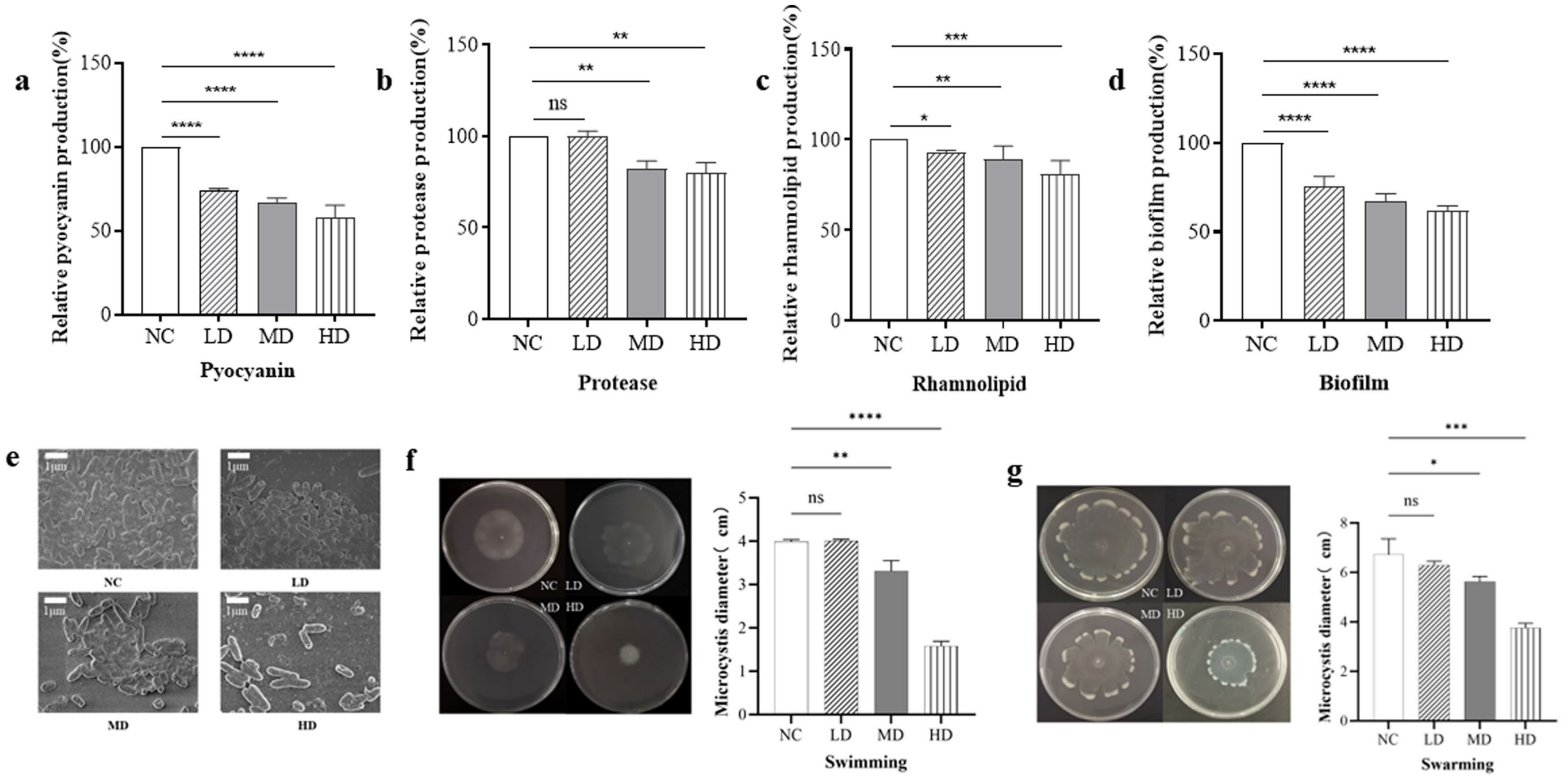
The effect of RmmLII on virulence factors production of PAO1. **(a)** Pyocyanin; **(b)** Extracellular proteases; **(c)** Rhamnolipids; NC, negative control. **(d)** Biofilm production; **(e)** The biofilm morphology under SEM; NC, negative control. **(f)** Swimming; **(g)** Swarming. NC: PAO1; LD: low dose group; MD: medium does group; HD: high dose group; ns, not significant; *p* > 0.05; *p < 0.05; **p < 0.01; ***p < 0.001;****p < 0.0001.

The effect of RmmLII on the secretion of PAO1 virulence factors was evaluated *in vitro*. Compared to the blank control group, the production of pyocyanin in the three experimental groups treated was significantly reduced (*p* < 0.0001), suggesting that RmmLII at varying concentrations can diminish pyocyanin expression by attenuating the QS system of *P. aeruginosa* PAO1. Among these, the high dose group treated with RmmLII exhibited the most significant reduction in pyocyanin expression (Figure 5a). Compared to the blank control group, the low dose group RmmLII had no significant effect on the production of extracellular proteases (*p* > 0.05), whereas the addition of medium and high does group RmmLII significantly inhibited extracellular protease production (*p* < 0.01) (Figure 5b). Compared to the blank control group, the rhamnolipid production in the three experimental groups showed varying degrees of reduction, with the high dose group demonstrating the most significant decrease, approximately 20% lower than the control group (Figure 5c). Although the decrease is modest, it is evident that the addition of RmmLII has a measurable impact on reducing rhamnolipid production. As the concentration of RmmLII increased, its inhibitory effect on the secretion of these three PAO1 virulence factors also progressively strengthened, showing a positive correlation between the concentration used and the intensity of inhibition.

### 3.7 Effect of RmmLII on the biofilm production *in vitro*

To evaluate the impact of varying concentrations of RmmLII on PAO1 biofilm formation (Figure 5d), a significant difference (*p* < 0.0001) was observed across all experimental groups compared to the blank control. RmmLII exhibited a notable inhibitory effect on biofilm formation in *P. aeruginosa* PAO1, with the most substantial impact observed at the high dose group. The biofilms formed by the different groups were observed by scanning electron microscopy (SEM). In the control group, the bacteria adhered firmly, forming a network-like structure between strains, with the biofilm densely covering the surface and exhibiting low heterogeneity. In the experimental groups treated with different concentrations of RmmLII, the biofilm was disrupted or cleared to varying extents. Particularly, in the high dose group, only a few bacteria remained, and the networked bacterial structure almost completely disappeared, resulting in near-total destruction of the biofilm structure (Figure 5e).

### 3.8 Effect of the motility of RmmLII *in vitro*

In the motility assay of PAO1, the swimming and swarming motility ranges were measured under the influence of RmmLII, followed by statistical analysis. RmmLII significantly inhibited both swimming and swarming motilities of PAO1 in the MD and HD groups (Figures 5f,g). Compared to the control group, swimming motility in the HD group was nearly completely abolished, demonstrating full inhibition (*p* < 0.0001). Similarly, swarming motility was markedly reduced, exhibiting a smaller range of activity and a more pronounced inhibitory effect (*p* < 0.001).

### 3.9 The survival rate of mice

**Figure 6:**
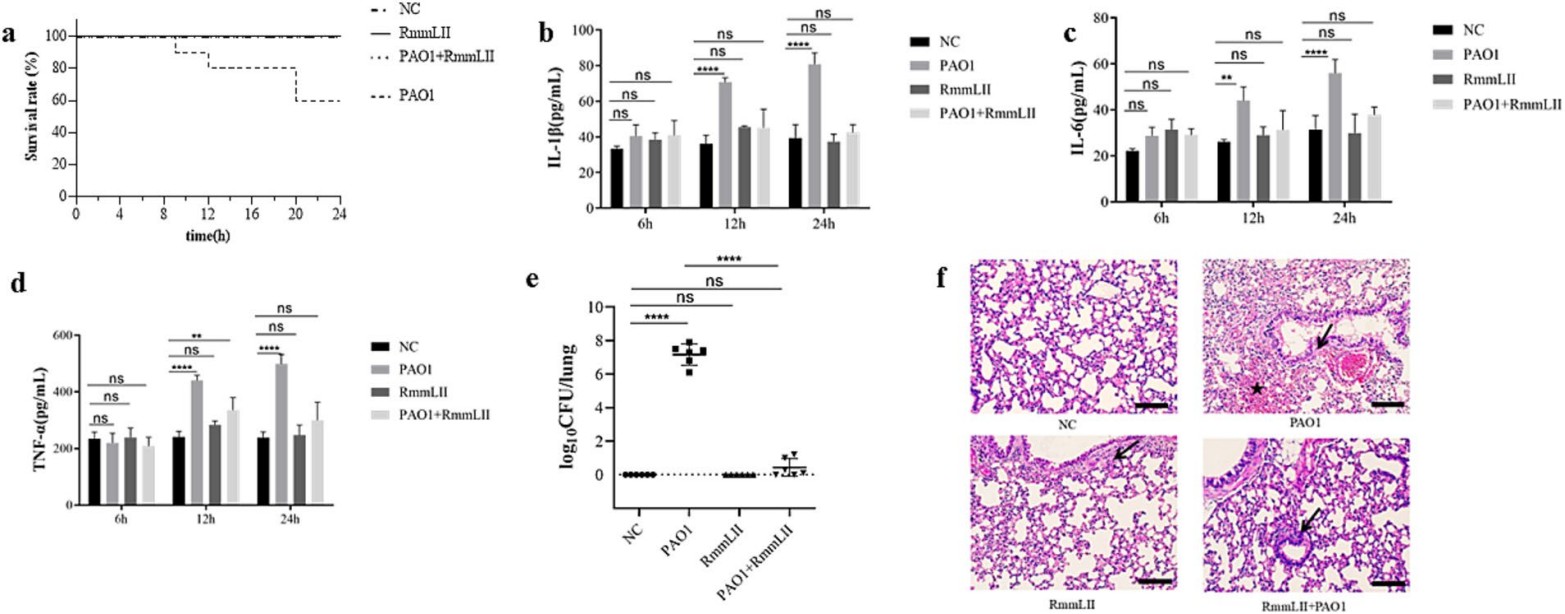
Evaluation of the *in vivo* inhibitory effect of RmmLII on PAO1 pathogenicity. **(a)** Mice survival rate (*n* = 10); **(b)** IL-1β; **(c)** IL-6; **(d)** TNF-α; **(e)** Lung bacterial load count; **(f)** Histological changes in the lungs of mice(200×). Scale bar, 100 = μm; ↙indicates reduced alveolar air space and infiltration of inflammatory cells; ★indicates bleed; ns, not significant; *p* > 0.05; *p < 0.05; **p < 0.01; ***p < 0.001; ****p < 0.0001.

BALB/c mice were administered 100 μL of RmmLII (1 mg/mL) intranasally 12 h before PAO1 infection, and the 24 h survival rate in the PAO1 + RmmLII group was 100%. In the PAO1 infection group, 4 mice succumbed, resulting in 24 h survival rate of 60%. No fatalities were observed in either the blank control group or the RmmLII group (Figure 6a). These results indicate that RmmLII can significantly reduce the mortality rate in mice following PAO1 infection.

### 3.10 Inflammatory factors and changes in bacterial load

Cytokines such as IL-1β, IL-6, and TNF-α play a central role in the body’s inflammatory response. Changes in their levels can accurately reflect the immune status and degree of inflammation of the body during the infection process of *P. aeruginosa* PAO1 (Chen et al., 2018; Gaspar et al., 2013). Detection of these cytokines can help us deeply understand the mechanism of the inflammatory response caused by infection and evaluate the intervention effect of RmmLII on the inflammatory response. Moreover, these cytokines are related to various diseases and are helpful for exploring potential therapeutic targets and strategies. ELISA results indicated that the cytokine levels of IL-1β, IL-6, and TNF-α in the serum of mice remained relatively stable at 6 h post-infection. After 12 h, the PAO1 model group showed a significant increase in IL-1β concentration (*p* > 0.0001), with gradual increases in IL-6 and TNF-α levels. At 24 h, the PAO1 model group exhibited markedly higher IL-1β and IL-6 levels (*p* > 0.0001), and a significant rise in TNF-α concentration (*p* < 0.05). No significant changes in cytokine levels were observed in the remaining groups (Figures 6b–d). PAO1 colony units from lung tissue homogenates 24 h post-infection were enumerated. No colonies were detected in the blank control and RmmLII groups, while the PAO1 group showed a significantly higher bacterial colony count compared to the PAO1 + RmmLII group. This finding suggests that RmmLII reduces PAO1 load in the murine lungs (Figure 6e), providing additional evidence for its effectiveness in acute lung infection in mice.

### 3.11 Histological analysis

Histopathological examination using HE staining revealed that when compared to the control group, the lungs of the PAO1 infection model group exhibited bruising, hemorrhage, and signs of epithelial cell degeneration and necrotic detachment. Erythrocytes and pale pink fluid were observed in the bronchioles, along with inflammatory cell infiltration in the alveolar lumens. In the PAO1 + RmmLII group, the epithelial lining of the terminal bronchioles remained intact, with minimal foreign body presence. However, some bronchiolar epithelial cells showed degeneration, accompanied by mild inflammatory cell infiltration and slight thickening of the alveolar walls. Overall, RmmLII mitigates the pathological changes induced by PAO1 infection in the murine lungs (Figure 6f).

## 4 Discussion

The widespread use of antibiotics has promoted the rise of antibiotic-resistant microorganisms, which poses a serious challenge to public health. The emergence of microbial resistance has led to a growing interest in the development of strategies for inhibiting QS systems. Recently, QQ enzymes have gained attention as a novel anti-QS strategy. QQ enzymes have their unique advantages, QQ enzymes can directly targets and degrade AHLs and interfere with bacterial communication without affecting the nontarget pathways. When compared with antibiotics, it reduces the risk of bacterial resistance. The broad-spectrum application to a variety of bacterial species regulated by QS makes it versatile in multiple scenarios.

Many marine bacteria have demonstrated QQ activity, and many types of QQ enzymes have been identified from marine species (Romero et al., 2012). In our previous studies (Cai et al., 2018), a novel AHL lactonase, RmmL, was identified and characterized from the YJ3 strain. This study confirmed the presence of another novel AHL lactonase, RmmLII, in YJ3, and characterized its properties. RmmLII shares a distinct amino acid sequence (HXHXDH) with other well-characterized AHL lactonases and is closely related to GKL from the PTE family, indicating a close evolutionary relationship. This is distinct from the previously characterized QQ enzyme RmmL identified in *T. mobilis* YJ3, which belongs to the metallo-β-lactamase family (Cai et al., 2018). The results from degradation activity assays and HPLC-MS analyses collectively demonstrate that RmmLII exhibits the capacity to hydrolyze AHL signal molecules, specifically degrading AHLs, thereby confirming its identity as an AHL lactonase, it exerts its function by catalyzing the hydrolysis of the lactone bond in signaling molecules. This mechanism of action is similar to that of other well-characterized AHL lactonases (Packiavathy et al., 2021).

Site-directed mutagenesis of RmmLII reveals that amino acids within the conserved domain may affect the enzyme’s activity. Previous studies have established that the conserved domain “HXHXDH” is essential for AHL lactone activity. In AiiA (Momb et al., 2008), site-directed mutagenesis experiments have demonstrated that His and Asp residues within the conserved domain are crucial for AiiA activity, while Tyr at position 194 forms a hydrogen bond between its phenolic side chain and the lactone carbonyl group of the substrate, thereby playing a pivotal role in substrate binding and catalysis. In MomL (Tang et al., 2015), site-directed mutagenesis was employed to replace His and Asp residues in the conserved domain with Ser, which lacks the capacity for metal binding, thereby demonstrating that the mutated amino acids significantly reduced the activity of MomL. This study demonstrates Pro82, Ala116, and Glu241 are essential for RmmLII activity and directly affect its degrading activity. The Glu241 in RmmLII is homologous to the Tyr194 in AiiA, a key residue directly involved in the catalytic mechanism (Charendoff et al., 2013; Tang et al., 2015). Furthermore, the mutation of glutamate to Gly198 in RmmLII enhances its degradation activity. This study emphasizes the significance of amino acids in highly conserved regions for RmmLII’s activity.

In terms of its biochemical properties, RmmLII exhibited good stability under various conditions. It maintained relatively stable activity between 20°C and 40°C and had an optimal reaction temperature of 60°C, similar to GKL from the thermophilic *Geobacillus kaustophilus*, a member of the same family (Chow et al., 2010). RmmLII also shows strong tolerance to alkaline environments, a trait not observed in other QQ enzymes such as Ahlx (Liu et al., 2019), MomL, and RmmL (Cai et al., 2018). Previous studies have demonstrated that, such as AiiA (Dong et al., 2000), Mn^2+^ and Cu^2+^ inhibit its activity, whereas Zn^2+^, Cu^2+^, and Mg^2+^ do not exert a significant effect on its activity. In contrast to AiiA, the addition of Zn^2+^, Cu^2+^, and EDTA, individually diminishes the activity of RmmLII, while Ca^2+^ and Mn^2+^ potentiate its activity. Addition of metal ions to EDTA-treated RmmLII demonstrated that EDTA can alleviate the inhibitory effect of Mn^2+^ on RmmLII activity. Investigations into the Mn-Mn dinuclear center within the GKL protein model complex suggest that lactonase activity relies on Mn^2+^ (Xue et al., 2013), which may imply that RmmLII is a metal-dependent enzyme. Further studies such as metal contents and kinetic constants of RmmLII will help to clarify the mechanism.

QQ enzymes can mitigate the pathogenicity and infectivity of specific gram-negative pathogens by inhibiting their physiological activities, including biofilm formation and toxin secretion (Fan et al., 2017; Migiyama et al., 2013; Sakr et al., 2021). The gene expression related to virulence factor secretion, biofilm formation, and motility of *P. aeruginosa* were regulated by AHL signaling (Chan et al., 2015). It has been demonstrated that *P. aeruginosa* PAO1 initiates colonization and attachment through swimming and swarming motilities and that QQ agents can decrease the attachment and colonization abilities that directly affect its biofilm-formation capacity (Gutierrez et al., 2013). The *in vitro* experiments demonstrated that RmmLII had a significant impact on the virulence factors of *P. aeruginosa* PAO1, such as extracellular proteases, pyocyanin, and rhamnolipid, which are the key virulence factors regulated by QS in the pathogen. RmmLII effectively inhibited the production of extracellular proteases, pyocyanin, and also showed a measurable reduction on rhamnolipid production. This is in line with the expected role of QQ enzymes in reducing bacterial virulence. Moreover, RmmLII showed a strong inhibitory effect on biofilm formation and motility of PAO1. The formation of biofilms represents a critical mechanism for bacterial survival and pathogenesis. Biofilms are microbial consortia that adhere to surfaces or aggregate with one another, encased in a self-produced extracellular matrix. Within biofilms, bacterial growth is protected against environmental challenges, including desiccation, mechanical forces, and immune system assaults (Rather et al., 2021). Consequently, disrupting biofilm formation is regarded as an effective strategy for combating pathogenic infections. The capacity of RmmLII to disrupt biofilm formation underscores its potential as a promising antibacterial agent. In mice models of acute lung infection, after 24 h of infection, the survival rate of mice pre-treated with RmmLII is 100%, while that of the control group is 60%. An analysis of lung bacterial load and pathological sections (Figures 6e,f) indicated that RmmLII provided nearly complete protection against infection and significantly improved survival rates. Furthermore, as we all know, QQ enzymes have the ability to downregulate the QS and virulence, but not to kill the pathogenic bacteria. In this research, it is very interesting that comparing to PAO1 group, there was much lower presence of PAO1 in the group treated with RmmLII enzyme. In our opinion, RmmLII interferes with the QS system of PAO1, effectively downregulating the virulence factors of PAO1. This reduces its ability to damage host tissues and makes it more easily recognized and eliminated by the host’s immune system, such as macrophages and neutrophils (Papenfort and Bassler, 2016). Secondly, RmmLII disrupts the formation of PAO1 biofilms, which are critical barriers that enable bacteria to resist the host immune system and antibiotics. The reduction in biofilm formation renders PAO1 more vulnerable to clearance by the immune system or antibiotics (Bjarnsholt et al., 2009). Furthermore, the QS system regulates the metabolic adaptability of bacteria. By inhibiting QS signaling, RmmLII may impair PAO1’s ability to effectively adapt to the host environment, including challenges such as nutrient competition and oxidative stress. This leads to disruption of bacterial growth and population dynamics, resulting in a reduction in their numbers (Ng and Bassler, 2009).

In this study, IL-1β, IL-6, and TNF-α were crucial. In the PAO1-infected mouse model, their concentrations increased significantly over time (Rather et al., 2022), indicating a robust inflammatory response. After RmmLII pre-treated, their secretion decreased significantly, demonstrating RmmLII’s effective intervention in the inflammatory process. IL-1β is essential for initiating and amplifying inflammation, and its increase in the model group corresponds with the activation of the inflammatory cascade. RmmLII’s inhibition of this process blocks excessive activation. The dual properties of IL-6 reflect the disrupted inflammatory balance during infection, and its reduction after treatment indicates a regulatory effect. The increase in TNF-α in the model group highlights the severity of infection-related inflammation, and the reduction of TNF-α by RmmLII demonstrates its ability to alleviate damage. Our study found that RmmLII reduced their secretion in the experimental group, potentially modulating the immune response in the pneumonia mouse model and mitigating acute lung infection, consistent with the findings on AiiM (Migiyama et al., 2013). Various QQ enzymes can interfere with the QS system *in vitro*, thereby reducing the pathogenicity of *P. aeruginosa*. For example, the administration of the lactonase SsoPox into the trachea of *P. aeruginosa* infected rats resulted in a significantly lower mortality rate in the treated group compared to the untreated group after a 50 h observation period (Hraiech et al., 2014). Similarly, the acylase PvdQ treatment resulted in a fivefold reduction in pulmonary bacterial load, decreased morbidity, alleviated tissue inflammation, and prolonged survival in the treated mice compared to the untreated control group (Utari et al., 2018). *N*-acyl homoserine lactonase can alleviate the intestinal disorders caused by *Salmonella typhimurium* infection in broiler chickens and improve the gut microbiota within the host (Wang et al., 2023). In conclusion, RmmLII holds great promise as a novel antibacterial agent for the prevention and treatment of bacterial diseases in animals. Future studies could focus on further optimizing its production and exploring its potential in combination with other antibacterial strategies. Additionally, in-depth investigations into its structure–function relationships could help to fully exploit its potential in combating bacterial infections and provide a solid foundation for the development of new antibacterial drugs targeting QQ enzymes.

## Data Availability

The datasets presented in this study can be found in online repositories. The names of the repository/repositories and accession number(s) can be found at: https://www.ncbi.nlm.nih.gov/, PP277415.

## Footnotes

^1^ https://www.uniprot.org/peptide-search
