# Supplementary figures and images for: RmmLII, a novel marine-derived N-acyl homoserine lactonase from Tritonibacter mobilis
Source: Front Microbiol. 2025 Mar 18;16:1538873. doi: 10.3389/fmicb.2025.1538873 (PMC11958948; doi:10.3389/fmicb.2025.1538873)

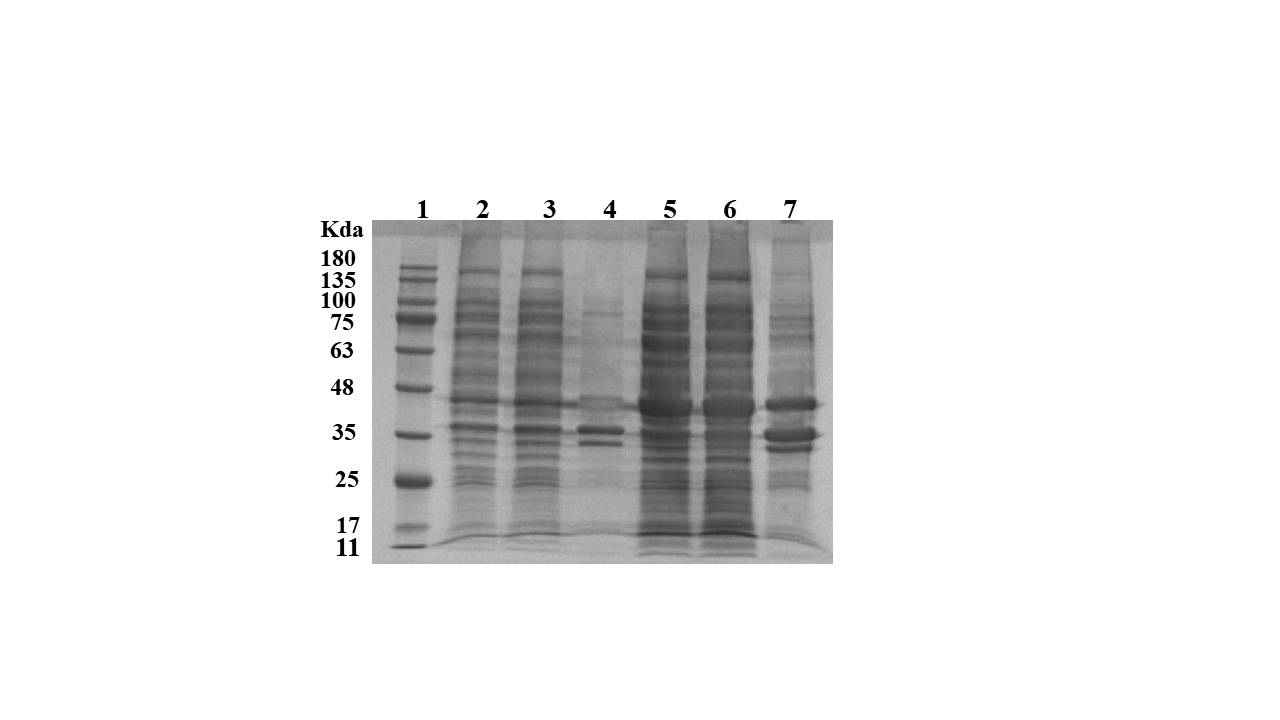

Supplement: Supplementary file 1 [file Data_Sheet_1.ZIP › 新建文件夹/Identification of recombinant bacteria for induced expression.tif]

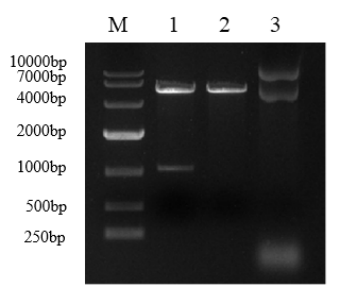

Supplement: Supplementary file 1 [file Data_Sheet_1.ZIP › 新建文件夹/Identification of recombinant plasmids by double enzyme digestion.tif]

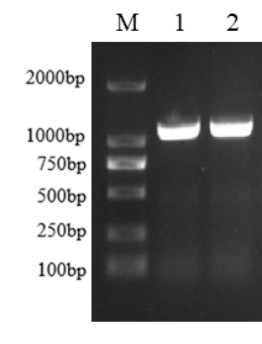

Supplement: Supplementary file 1 [file Data_Sheet_1.ZIP › 新建文件夹/PCR amplification of RmmLII genes.tif]

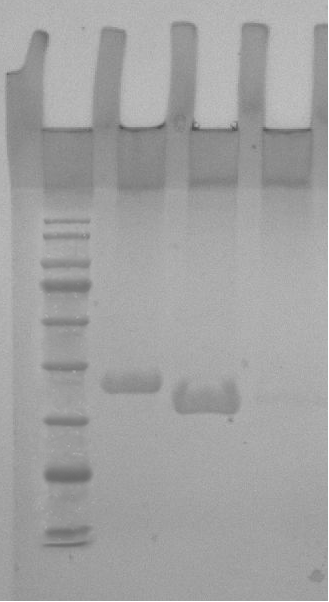

Supplement: Supplementary file 2 [file Image_1.TIF]

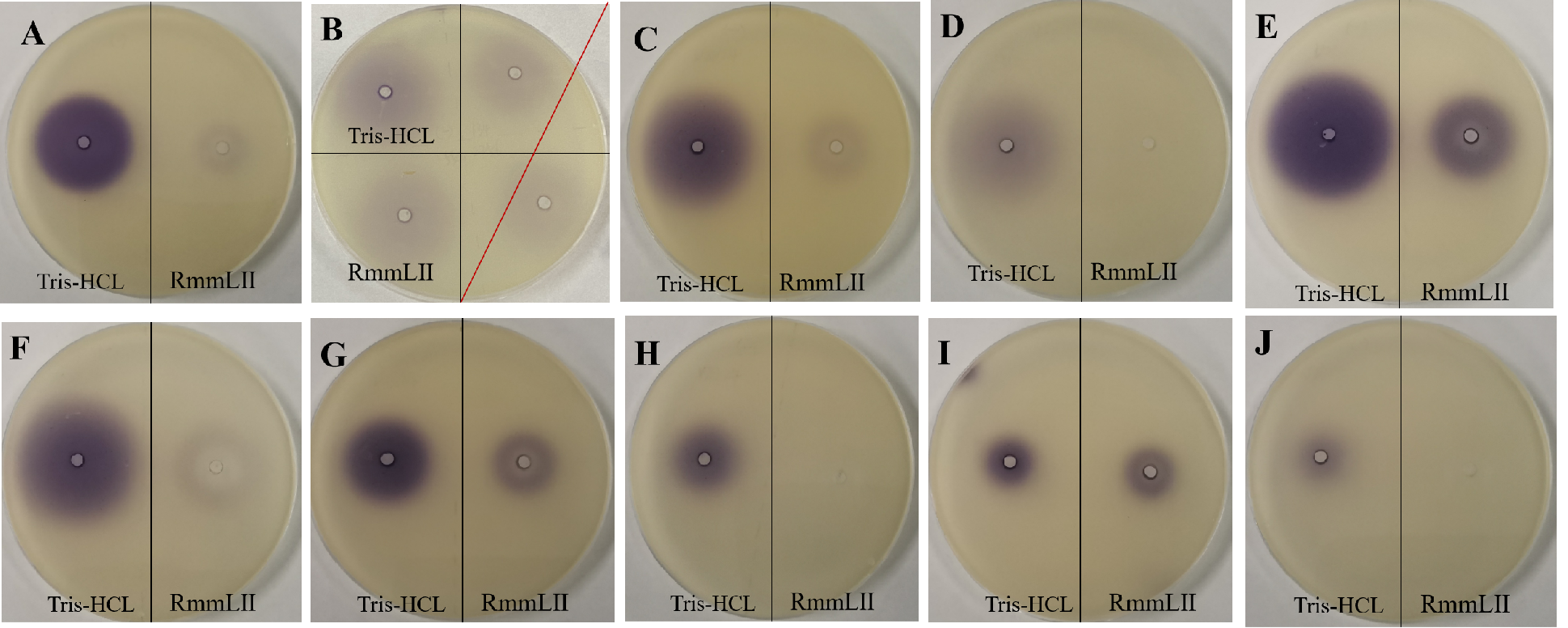

Supplement: Supplementary file 3 [file Image_2.TIF]

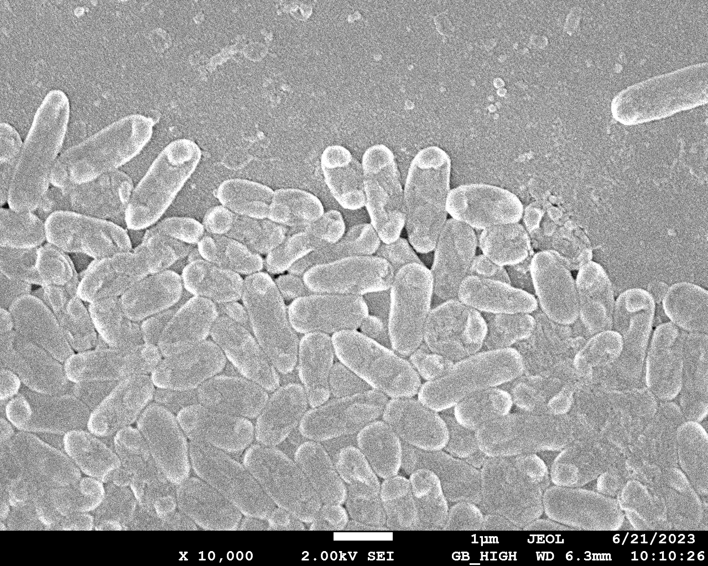

Supplement: Supplementary file 4 [file Image_3.TIF]

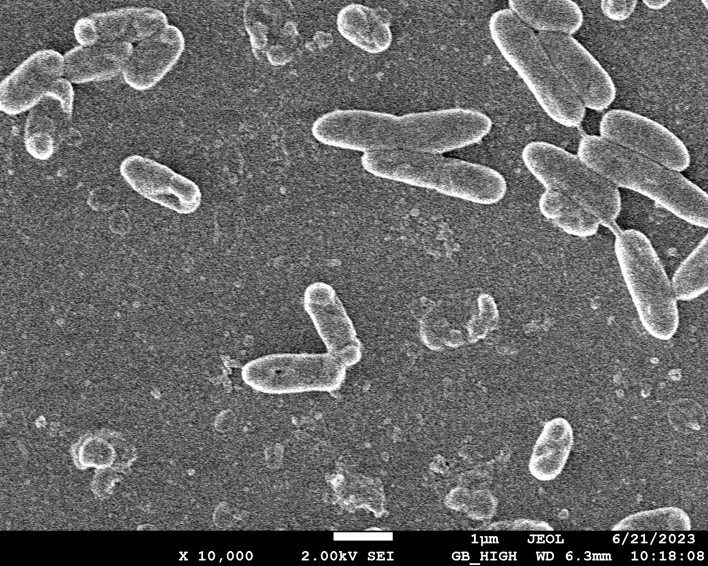

Supplement: Supplementary file 5 [file Image_4.TIF]

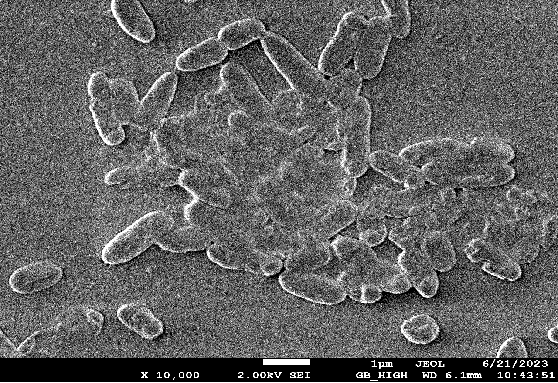

Supplement: Supplementary file 6 [file Image_5.TIF]

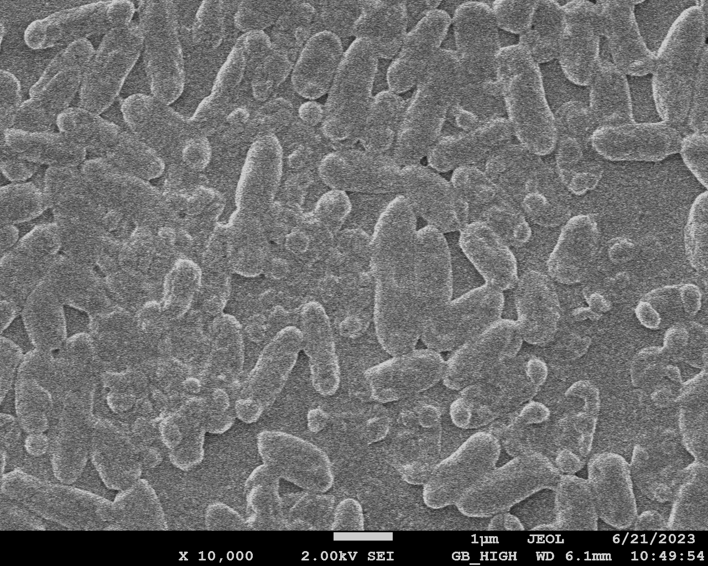

Supplement: Supplementary file 7 [file Image_6.TIF]

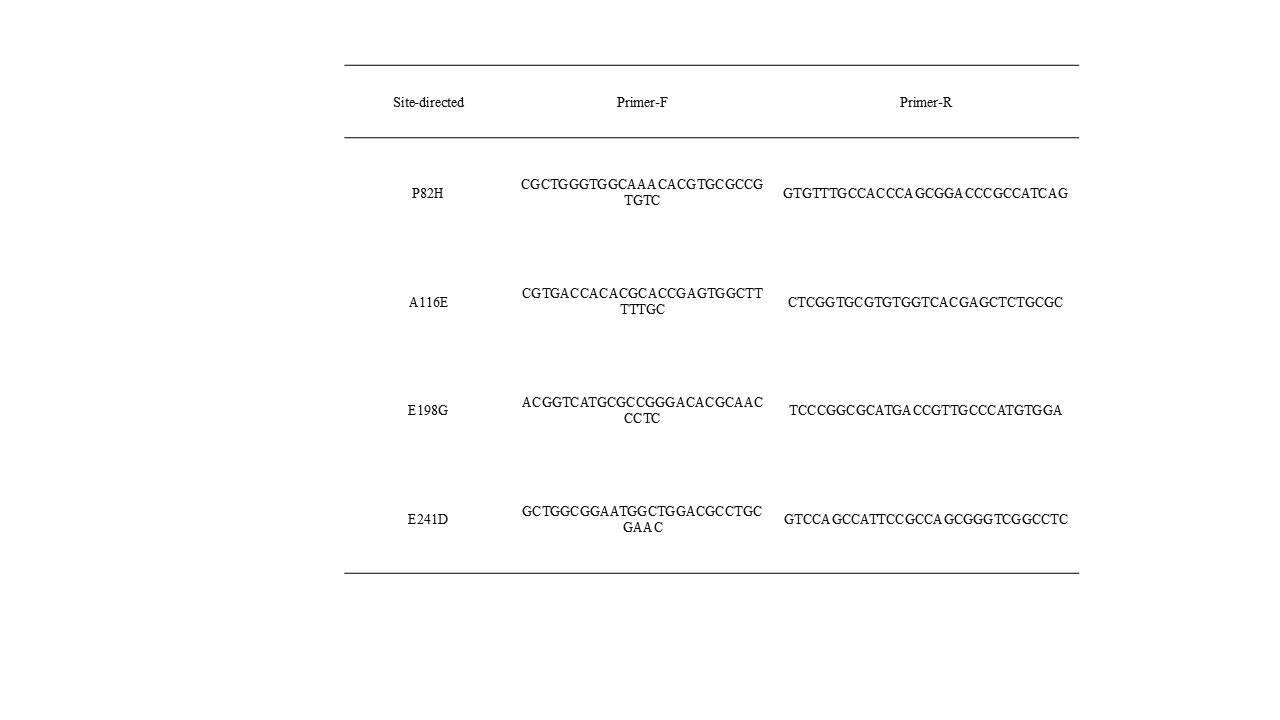

Supplement: Supplementary file 8 [file Image_7.TIF]
